# Comparing efficacy and safety of oral drugs in treatment of hyperthyroidism: a systematic review and network meta-analysis

**DOI:** 10.7717/peerj.20403

**Published:** 2026-01-28

**Authors:** Hui Zhang, Minghao Lin, Peng Zhang, Dexi Zhao, XiangYue Ma, Yujuan Fu

**Affiliations:** 1 Changchun University of Chinese Medicine, Changchun, China; 2 The Affiliated Hospital to Changchun University of Chinese Medicine, Changchun, Jilin, China

**Keywords:** Hyperthyroidism, Network meta-analysis, Randomized controlled trial, Free triiodothyronine, Free thyroxine, Thyroid stimulating hormone

## Abstract

**Background:**

Thyrotoxicosis refers to a condition where there is an excess of thyroid hormone production by the thyroid gland itself, leading to a form of hyperthyroidism.

**Objective:**

This study is based on systematic review and network meta-analysis methods, aiming to provide a more reliable basis for the selection of clinical treatment plans by comprehensively considering the efficacy and safety of different drugs in treating hyperthyroidism, including the regulation of thyroid hormone levels and potential adverse reactions.

**Methods:**

Computerized searches were conducted in eight major domestic and international databases (PubMed, Cochrane Library, Embase, Web of Science, CBM, CNKI, WANFANG DATA, VIP) for randomized controlled trials (RCTs) on the efficacy of oral medications in improving the treatment outcomes of patients with hyperthyroidism. The search period covered from the inception of each database to October 2025. All researchers independently screened the literature, extracted data, and assessed the quality of the studies. For studies meeting the quality criteria, data analysis was performed using Stata 17.0 and RevMan 5.4 software.

**Results:**

A total of 151 articles were ultimately included, involving 14,158 patients, with 7,084 in the treatment group and 7,074 in the control group. The network meta-analysis showed that in terms of total effective rate, the top three interventions with the highest Surface Under the Cumulative Ranking Curve (SUCRA) probability ranking curve area were I-131 (Iodine-131)+LC (lithium carbonate), MMI (Methimazole)+PHT (Propranolol), and I-131; in terms of reducing Free Triiodothyronine (FT3), the top three interventions with the highest SUCRA probability ranking curve area were MMI+PDN (prednisone), I-131+LC, and I-131; in terms of reducing free tetraiodothyronine (FT4), the top three interventions with the highest SUCRA probability ranking curve area were I-131+PTU (Propylthiouracil), I-131+LC, and I-131; in terms of increasing thyroid-stimulating hormone (TSH), the top three interventions with the highest SUCRA probability ranking curve area were I-131+PTU, I-131, and MMI+PDN; in terms of reducing adverse reactions, the top three interventions with the highest SUCRA probability ranking curve area were I-131+PTU, I-131, and I-131+MMI.

**Conclusion:**

This study indicates that among the interventions for treating hyperthyroidism, I-131+LC has a higher efficacy rate compared to other treatments, I-131+PTU is superior in reducing FT3, increasing TSH and reducing adverse reactions compared to other treatments. Due to the limitations in the quantity and quality of the included studies, the aforementioned conclusions await further validation from more large-sample, high-quality, and multicenter studies. PROSPERO study number CRD42024566298.

## Introduction

Hyperthyroidism refers to a condition of thyrotoxicosis caused by the overproduction of thyroid hormones by the thyroid gland itself (Ross et al., 2016). It has a global prevalence of 0.2–1.3% and is a disease that often affects multiple systems and organs, potentially impacting life. Therefore, early diagnosis and rational medication can effectively reduce the harm caused by hyperthyroidism. In terms of pathogenesis, autoimmune factors are one of the key causes of hyperthyroidism (Antonelli et al., 2020). In autoimmune hyperthyroidism, the immune system malfunctions and produces specific antibodies against the thyroid-stimulating hormone receptor thyroid-stimulating hormone receptor (TSHR) on the surface of thyroid cells, namely thyroid-stimulating hormone receptor antibodies thyrotropin receptor antibody (TRAb) (Petranović Ovčariček, Görges & Giovanella, 2024). Among them, the stimulating TRAb can bind to TSHR, mimicking the action of thyroid stimulating hormone (TSH), continuously activating the signaling pathways within thyroid cells, and causing uncontrolled increases in the synthesis and release of thyroid hormones, ultimately leading to hyperthyroidism. Additionally, other autoantibodies such as thyroid peroxidase antibodies TPOAb and TgAb may also participate in disrupting the normal structure and function of thyroid cells, further exacerbating thyroid dysfunction (Wiersinga, Poppe & Effraimidis, 2023).

Currently, the treatment options for hyperthyroidism are mainly divided into surgical and non-surgical treatments. Non-surgical treatments include I-131, thiourea drugs, and combination therapies with beta-blockers or hormonal medications. Surgical treatments still have controversies regarding the extent of thyroidectomy, with main methods including total thyroidectomy, near-total bilateral thyroidectomy, subtotal bilateral thyroidectomy, and hemithyroidectomy plus subtotal contralateral thyroidectomy (Al-Shakhrah, 2008). Antithyroid drugs inhibit the formation and coupling of iodotyrosine in thyroglobulin, which is essential for thyroid hormone synthesis (Li, Teng & Ba, 2020). In China, antithyroid drugs (ATDs) are used as the first-line treatment for hyperthyroidism. Although methimazole has been widely used for a long time, its use is often limited by its systemic adverse reactions (Dongdong & Xiaohua, 2021). Current oral drug treatments for hyperthyroidism have their respective advantages and adverse reactions. Special attention should be paid to hyperthyroidism patients with pregnancy, COVID-19, or other complications (such as atrial fibrillation, thyrotoxic periodic paralysis, and thyroid storm). Hyperthyroidism is associated with increased mortality. Therefore, this study, based on network meta-analysis, evaluates the clinical efficacy and safety of different drugs for hyperthyroidism, aiming to provide objective evidence-based support for clinical medication and further develop better research methods for different indicators in treating hyperthyroidism.

## Material and Methods

The study has been registered with the International Prospective Register of Systematic Reviews (PROSPERO) under study number CRD42024566298. This network meta-analysis study was conducted in strict accordance with guidelines based on the Preferred Reporting Program for Systematic Review and Meta-Analysis (PRISMA).

### Search strategy

Nine randomized controlled experiments for the treatment of hyperthyroidism were searched through eight databases (PubMed, Web of Science, Embase, Cochrane Library, CNKI, WANFANG, VIP, CBM) up to October, 2025, with a blind method, a year of publication, and no language restriction. Search by combining medical subject heading (network) terms and free words, “Hyperthyroidism”, “Hyperthyroid”, “Hyperthyroids”, “Primary Hyperthyroidism”, “Hyperthyroidism, Primary”, “131I-TM-601”, “propylthiouracil ”, “Methimazole”, “Propranolol”, “Lithium Carbonate”, “ Prednisone”. PubMed Search strategy is shown in Supplement 1.

### Research type

This study is based on systematic review and network meta-analysis methods, aiming to provide a more reliable basis for the selection of clinical treatment plans by comprehensively considering the efficacy and safety of different drugs in treating hyperthyroidism, including the regulation of thyroid hormone levels and potential adverse reactions. Published randomized controlled trials (RCTs), with no language restrictions.

### Study population

Patients diagnosed with hyperthyroidism, with diagnostic criteria referring to the Writing Group of the Chinese Guidelines for the Diagnosis and Treatment of Thyroid Diseases & Endocrine Society of Chinese Medical Association (2007) or the Kahaly et al. (2018). RCTs of all original studies, irrespective of source or country, published in English or Chinese language only. Adult (18–75 years old) patients diagnosed with hyperthyroidism, regardless of gender, and course of the disease.

### Intervention measure

Experimental group: MMI, I-131, PHT, PTU, PDN, LC. The dosage and duration of treatment are not restricted. The above drugs can be used either alone or in combination.

Control group: MMI, I-131, PHT, PTU, PDN, LC. The dosage and duration of treatment are not restricted. The above drugs can be used either alone or in combination.

The dosages of various medications are as follows: PTU 50 mg/day, with a reasonable reduction in dosage as the patient’s condition improves, ultimately maintaining a controlled dose of two mg/day. One treatment course consists of continuous medication for 2 months. MMI 15–60 mg/day. The dosage of I-131 is calculated as 65 µCI per gram of thyroid tissue, specifically administering I-131 (sodium iodide (I-131) oral solution, produced by Atom High Tech Co., Ltd., National Medicine Approval No. H10960250) 7,400 MBq, averaging 5.5 mCI, taken orally in a single dose by Utheisa Kong. Tapazole: Oral administration, 10 mg each time, 3 times/day, followed by adjustment of the dosage to 10 mg/day as a single dose once thyroid hormone levels return to normal. PDN: Oral administration, 10 mg each time, 3 times/day, followed by adjustment of the dosage to 10 mg/day as a single dose once thyroid hormone levels return to normal. Propranolol: 10 mg/day.

### Outcomes

① Total effective rate; ② FT3; ③ FT4; ④ TSH; ⑤ adverse effects.

### Exclusion criteria

Randomized controlled trials that meet one of the following conditions are excluded: ① Randomized controlled trials that do not meet the evaluation criteria of clinical efficacy; ② Repeat the report; ③ Interventions involving Chinese herbal medicine, acupuncture or other drug combination treatment; ④ No correlation outcome; ⑤ The full text of the study is not available; ⑥ The data is incorrect or incomplete.

### Data extraction

All articles are managed by Endnote software and completed by two people (Hui Zhang and Minghao Lin). In case of disputes, they are discussed by Yujuan Fu. Selection is made by excluding irrelevant articles, reviews, and animal experiments. Two evaluators used Excel (Microsoft, Redmond, WA, USA) to independently extract the eligible study data, collecting the following information: sample size, gender, age of each group; Interventions (types and duration of oral drugs); Outcome indicators: ① Effective rate; ② FT3; ③ FT4; ④ TSH; ⑤ Adverse reactions.

### Quality assessment

Methodological quality risk assessment of the included randomized controlled trials was assessed through the Cochrane Risk Bias Assessment Tool 5.4.1 with the following items: random sequence generation, allocation concealment, subject and researcher blindness, outcome assessment blind, incomplete outcome data, selective reporting, and other bias. The risk of bias is completed by two persons (Hui Zhang and Minghao Lin), and in case of disagreement, it will be discussed by the third party Yujuan Fu. Apply RevMan 5.4.1 plots for quality assessment.

### GRADE quality of evidence

The online GRADEproGDT software system is used to conduct the GRADE grading system evaluation of the quality of evidence for outcome indicators (https://www.gradepro.org/). The quality of evidence is categorized into four levels: high, moderate, low, and very low. The evaluation of the quality of evidence for outcome indicators is based on five factors: risk of bias, inconsistency, indirectness, imprecision, and publication bias, resulting in the formation of a GRADE evidence summary table.

### Statistical analysis

The statistics for the network meta-analysis are based on a frequentist framework, with all outcome indicators analyzed using a random-effects model. For continuous variables, the standardized mean difference (SMD) is used, while for dichotomous variables, the odds ratio (OR) is used as the combined effect size, with each effect size represented by a 95% confidence interval (CI). Stata 17.0 software is used to conduct the network meta-analysis within a frequentist framework, employing the network command for data preprocessing and to plot the network relationships between interventions for each outcome indicator. When closed loops are formed among interventions, an inconsistency test is conducted to assess the consistency between direct and indirect comparison results. The surface under the cumulative ranking curve (SUCRA) is used to rank the probability of therapeutic effects among interventions, identifying the relatively optimal intervention. ReviewManager 5.4.1 software is used for literature quality assessment.

### Data synthesis

During the data collection phase, we conducted a comprehensive search of the literature, covering eight authoritative databases. Based on the established inclusion and exclusion criteria, we screened 3,419 articles, 151 articles were included. For the data of the included studies, we performed meticulous preprocessing, including checking the completeness and accuracy of the data, filling in missing values using appropriate methods, and identifying and handling outliers to ensure data quality and lay a solid foundation for subsequent synthesis.

For studies suitable for inclusion in the network meta-analysis (NMA), we used STATA 17.0 to standardize the data from different studies, unify the calculation methods and measurement units of effect sizes to ensure comparability of the data. During the analysis, we fully considered the heterogeneity between studies and used a random-effects model for analysis to make the results more robust. At the same time, we used visualization tools such as network diagrams, SUCRA, and league tables to intuitively display the effect sizes and their confidence intervals of each study for evaluation.

## Result

### Literature retrieval

**Figure 1:**
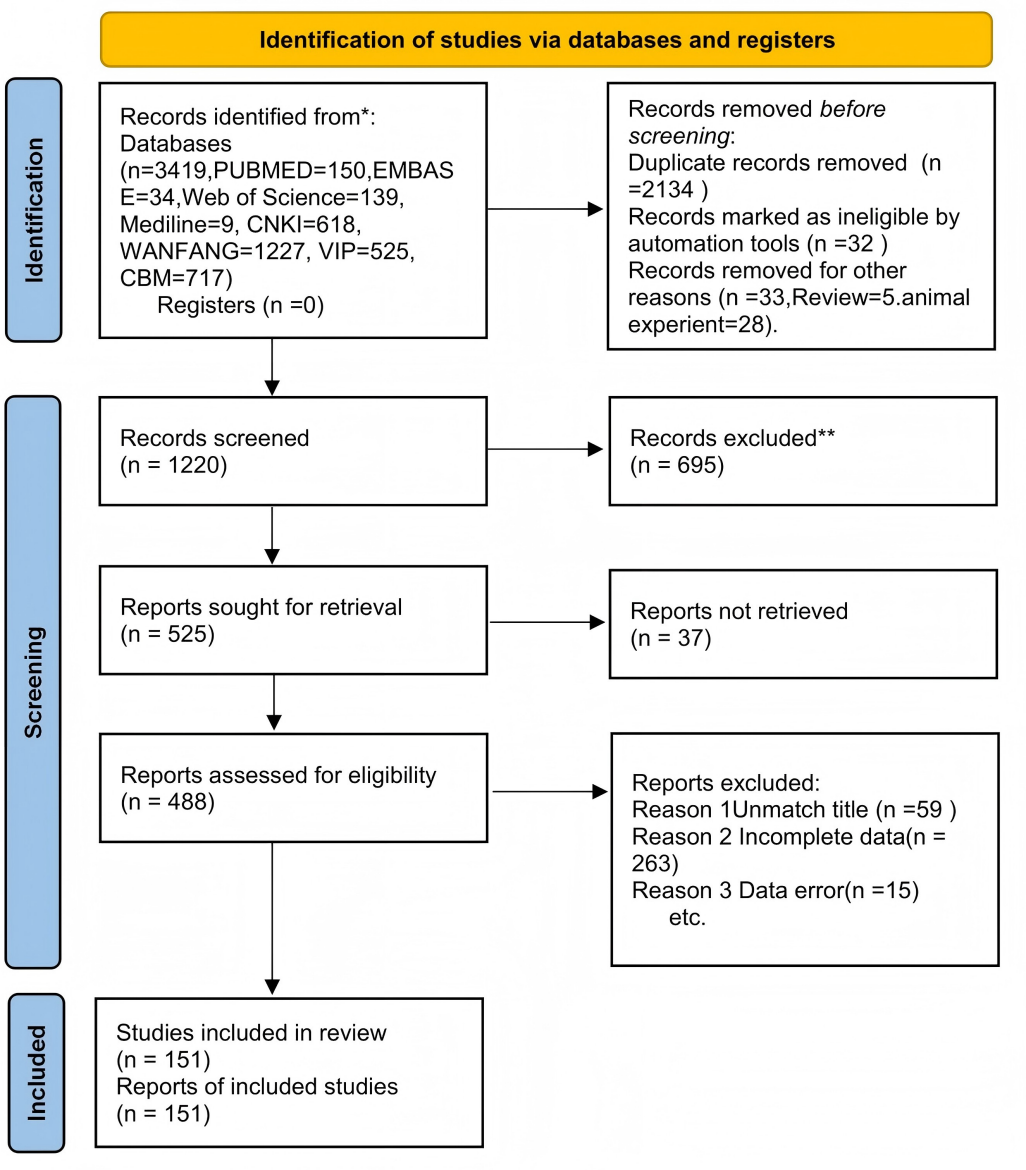
Flow diagram.

A total of 151 articles (Wen, Xiangzeng & Yadi, 2021; Yao, 2023; Zhenzhen, 2019; Yeli, 2019; Wenju, 2020; Jing, 2018; Liang, 2021; Jinqiu, 2018; Lingsu, 2020; Jinfeng, 2018; Zhang, Gao & Yang, 2016; Chen, Sui & Feng, 2019; Changqian, 2019; Su, 2019; Wang, 2018d; Li & Ning, 2016; Wang, 2018c; Wenfang, 2019; Xiaoqin, Guangtao & Yajun, 2019; Xiu, 2021; Cui, 2021; Liang & Jing, 2021; Liu, 2019; Yoxia, 2018; Jianjian & Shuai, 2019; Xue, 2021; Liping, Hongmei & Youwei, 2018; Quansheng & Zhao, 2020; Xiaoli, 2019; Zhiming, 2019; Wu, 2020; Jing, 2020; Wei & Binhong, 2019; Yabin, Hui & Quanwu, 2016; Yujuan, Gang & Haiyan, 2018; Sun, 2018; Mengting, Cuishang & Weijing, 2021; Yao, 2021; Yonghui, 2020; Weiwei, 2023; Shuai, 2018; Zhang, 2019; Li, 2017; Chang, 2019; Xiang, 2014; Chang & Guangyu, 2021; Li, 2019; Suyan & Qianya, 2021; Jing, Lili & Chao, 2021; Zhang, 2018; Shuiyan & Guoping, 2016; Ran, Caihong & Qingmei, 2019; Canqing, 2022; Min, Nai-qiang & Hua, 2016; Chaoxia, 2019; Yongwu, 2024; Yuan, 2019; Chen, 2021; Weifeng, 2012; Qiuying, 2021; Wenchao, Cailiang & Rensen, 2012; Cailing, 2017; Jing & Zhe, 2015; Ling, Zhihua & Yuanyuan, 2018; Liping, Jia & Jingxian, 2017; Guihong, 2020; Bo, Rongli & Lei, 2018; Na, 2019; Wang, 2018b; Ying, Yang & Xiaoxiao, 2019; Yunxiang, Xingming & Wanjun, 2002; Caifeng, 2019; Xu, 2018; Yishu, 2023; Yanfei & Yan, 2024; Jiayuan & Huimin, 2021; Jiaxin & Yanyun, 2021; Yilin, 2012; Wang, 2021; Xuehui, Qingfeng & Yun, 2019; Yi, 2018; Bing, 2016; Youyin, 2019; Cao, 2021b; Guilin & Lichao, 2019; Huiyu & Quanzhen, 2021; Siming & Ruojia, 2021; Yongming, Ming & Xiaochun, 2017; Na, 2023; Ping, Xiaoli & Qian, 2019; Mei, 2012; Xiaofeng, 2018; Yuan, 2022; Xueping, Qinghua & Haisheng, 2023; Wei & Jiao, 2019; Chenxi, 2002; Ding, 2017; Baolin, 2017; Li-ming, Li & Wei-wei, 2022; Pan, 2021; Liu, 2018; Xianfeng, 2019; Nai-lin, 2022; Lixian, 2022; Xueping, 2019; Lijuan, 2018; Xiao, Yongcheng & Chiquan, 2019; Yingying, 2021; Wang, 2020; Yue, Xiangru & Delong, 2017; Rendong, 2017; Zhou, 2016; Fengjuan, 2016; Hongju & Dongping, 2019; Wang, 2018a; Yilin, 2019; Junhui, 2020; Jiadong, 2019; Hao & Guimei, 2016; Che, 2017; Da, 2013; Cao, 2021a; Gai & Junlin, 2021; Xi, 2002; Jine & understood as Ci, 2019; Zhuo, 2012; Dongfei, Huan & Yi, 2022; Lihua, 2021; Huali, 2018; Yan-Hui, Yun-Peng & Yuan-zhi, 2018; Xianjing, 2019; Tingting, Wei & Xinjian, 2023; Li, 2023; Guiping, 2016b; Qiuya, 2021; Ye, 2022; Ai, Jing & Xuan, 2022; Yadong & Yanni, 2023; Rendi, 2019; Zuliang, 2019; Fanghua, 2020; Wan, Yuping & Qidong, 2018; Zheng, 2022; Wanmei, Jiaqi & Wanwen, 2021; Guiping, 2016a; Hongying, 2017; Xiong, 2018; Rui & Jing, 2021; Hu et al., 2024; Ducheng, 2025) with a total of 14,158 patients were included, including 7084 patients in the experimental group and 7074 patients in the control group. The therapeutic measures included in this article are as follows: MMI, I-131, PHT+MMI, PTU, I-131+PTU, I-131+MMI, MMI+PDN, PDN+PTU, I-131+LC. The flow diagram is shown in Fig. 1, and the basic information of the included literature is in Supplement 2.

### Quality of the included literature

**Figure 2:**
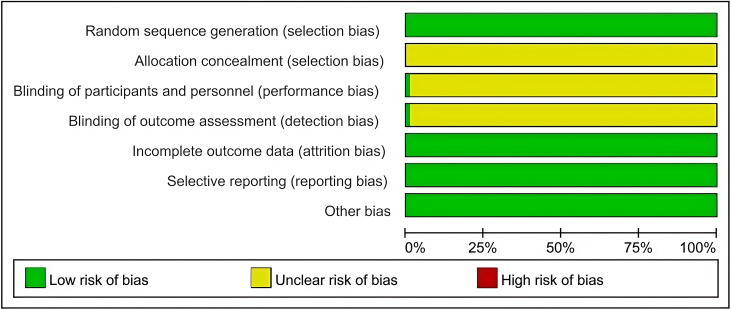
Risk of bias graph.

In the 151 included articles, baseline data were comparable between the test and control groups. 151 items were assigned by random method as “low risk”; or “unclear risk”. Two reported blind use, rated as “low risk”. All literature outcome data were complete and no selective reporting bias was found, rated as “low risk”. Other sources of bias were unclear and all were rated as uncertain. The risk of bias evaluation is shown in Fig. 2.

### Outcomes of network meta-analysis

#### Effective rate

##### Evidence network diagram.

**Figure 3:**
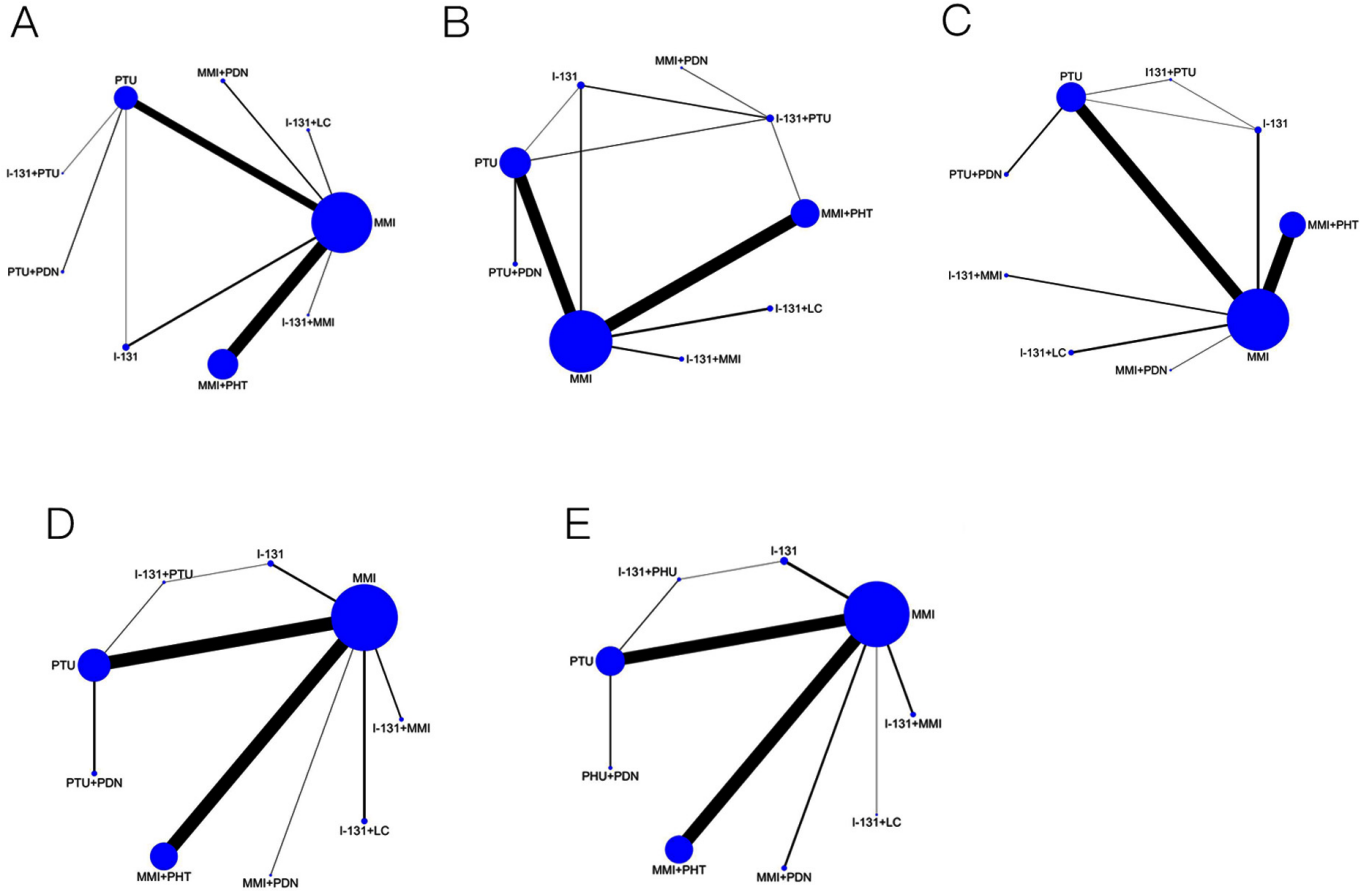
Network diagram. (A) Effective rate evidence network diagram, (B) FT3 evidence network diagram, (C) FT4 evidence network diagram, (D) TSH evidence network diagram, (E) Adverse reactions evidence network diagram.

A total of 103 RCTs reported the total effective rate, involving nine types of Western medicine, with the network centered around MMI. The size of the circles represents the sample size of the interventions, indicating that MMI has the largest volume of literature and the greatest sample size. A closed loop is formed, so an inconsistency test is conducted, with *P* > 0.05, indicating that the data generally meet the consistency test. See Fig. 3A for details.

##### SUCRA probability ranking plot.

**Figure 4:**
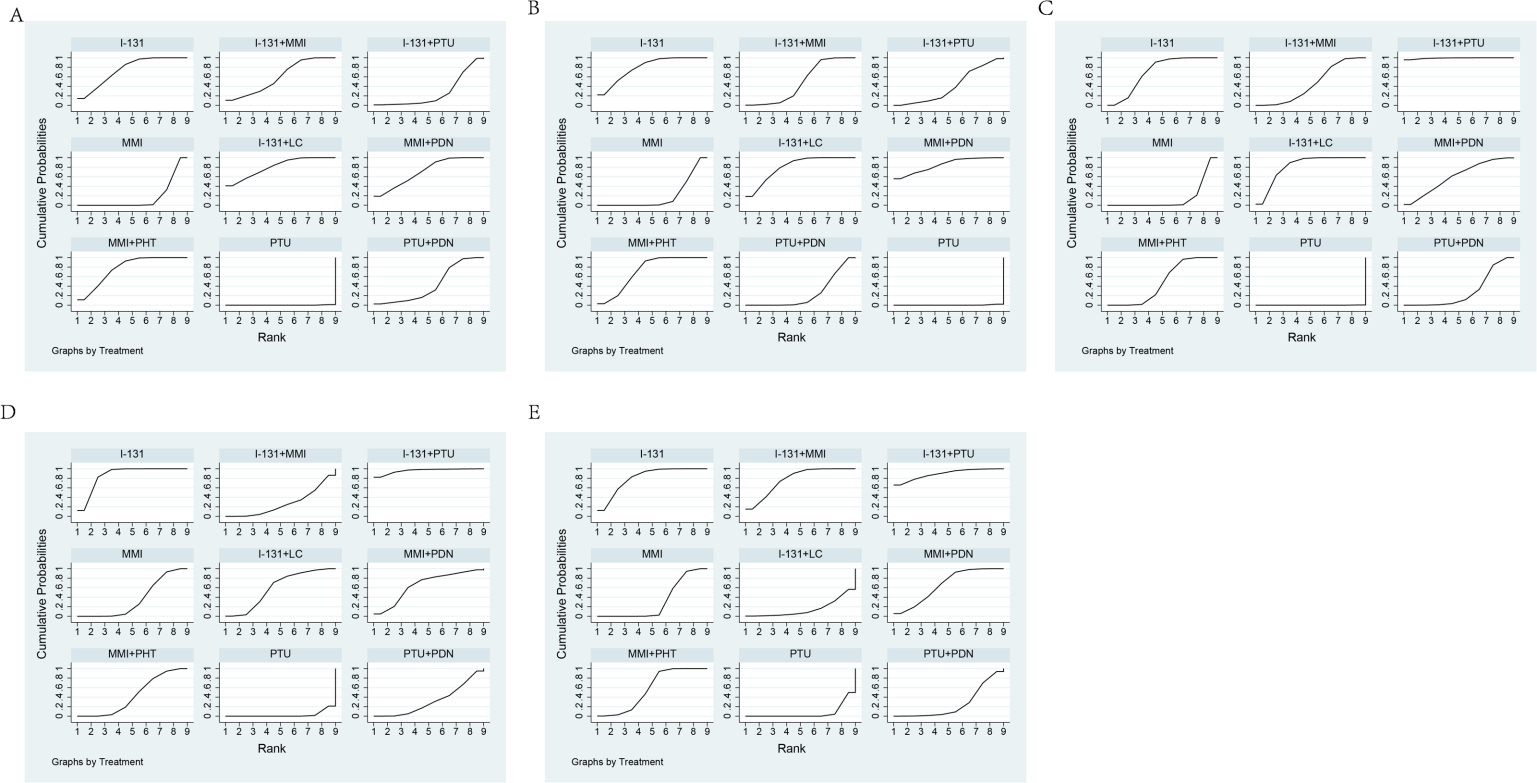
SUCRA probability ranking plot. (A) Effective rate SUCRA probability ranking plot, (B) FT3 SUCRA probability ranking plot, (C) FT4 SUCRA probability ranking plot, (D) TSH SUCRA probability ranking plot, (E) Adverse reactions SUCRA probability ranking plot.

In terms of the total effective rate, the SUCRA probability ranking diagram is I-131+LC >MMI+PHT >I-131 >MMI+PDN >I-131+MMI >PTU+PDN >I-131+PTU >MMI >PTU, indicating that I-131+LC has the best curative effect in the treatment of hyperthyroidism, as shown in Fig. 4A.

##### League table.

A network meta-analysis was performed on the included studies, and a total of 18 pairwise comparisons were generated. The results showed that the differences were statistically significant (*p* < 0.05) as follows: MMI + PHT was superior to I-131 + PTU. See Table 1.

**Table 1:** League table of effective rate.

Intervention measure	I-131+LC	MMI+PHT	I-131	MMI+PDN	I-131+MMI	PTU+PDN	I-131+PTU	MMI	PTU
I-131+LC	0								
MMI+PHT	0.10 (−0.71, 0.91)	0							
I-131	0.12 (−0.75, 1.00)	0.03 (−0.44, 0.49)	0						
MMI+PDN	0.17 (−0.85, 1.20)	0.08 (−0.63, 0.79)	0.05 (−0.73, 0.83)	0					
I-131+MMI	0.39 (−0.77, 1.56)	0.30 (−0.60, 1.19)	0.27 (−0.68, 1.22)	0.22 (−0.87, 1.31)	0				
PTU+PDN	0.81 (−0.46, 2.08)	0.71 (−0.31, 1.74)	0.69 (−0.39, 1.76)	0.64 (−0.57, 1.84)	0.42 (−0.91, 1.74)	0			
I-131+PTU	1.43 (−0.08, 2.93)	1.33 (0.02, 2.64)	1.30 (−0.05, 2.65)	1.25 (−0.20, 2.71)	1.03 (−0.52, 2.58)	0.62 (−0.98, 2.21)	0		
MMI	0.95 (−0.05, 1.95)	1.66 (1.43, 1.89)	1.63 (1.23, 2.04)	1.36 (0.50, 2.23)	0.33 (−0.96, 1.62)	1.58 (0.91, 2.26)	−1.10 (−1.34, −0.86)	0	
PTU	2.86 (2.05, 3.67)	2.76 (2.43, 3.09)	2.74 (2.27, 3.20)	2.69 (1.97, 3.40)	2.47 (1.57, 3.36)	2.05 (1.08, 3.02)	1.44 (0.17, 2.70)	0.74 (−2.15, 3.64)	0

#### FT3

##### Evidence network diagram.

FT3 was reported in 144 RCTS, involving nine therapeutic measures. The network relationship was centered on MMI, and the size of the dots represented the sample size of the interventions, indicating that MMI had the largest number of literatures and the largest sample size. There is a closed loop, so the inconsistency test is carried out, *P* > 0.05, the data basically meet the consistency test. See Fig. 3B for details.

##### SUCRA probability ranking plot.

On FT3, the SUCRA probability sequence is MMI+PDN > I-131+LC > I-131 > MMI+PHT > I-131+MMI > I-131+PTU > PTU+PDN > MMI > PTU, indicating that MMI+PTU is the best way to reduce FT3. See Fig. 4B.

##### League table.

A network meta-analysis was performed on the included studies, and a total of 18 pairwise comparisons were generated. The results showed that the differences were statistically significant (*p* < 0.05) as follows: MMI+PDN was superior to I-131+PTU, PTU; WM; I-131+LC is superior to PTU+PDN, MMI, PTU; I-131 is superior to PTU+PDN, MMI, PTU; MMI+PHT is better than PTU+PDN, MMI, PTU; I-131+PTU is superior to PTU, and PTU+PDN is superior to PTU. See Table 2.

**Table 2:** League table of FT3.

Intervention measure	MMI+PDN	I-131+LC	I-131	MMI+PHT	I-131+MMI	I-131+PTU	PHU+PDN	MMI	PTU
MMI+PDN	0								
I-131+LC	−0.80 (−5.20, 3.61)	0							
I-131	−0.81 (−5.34, 3.71)	−0.02 (−2.21, 2.18)	0						
MMI+PHT	−1.21 (−5.36, 2.94)	−0.41 (−1.90, 1.07)	−0.40 (−2.20, 1.41)	0					
I-131+MMI	−2.50 (−6.96, 1.96)	−1.70 (−3.76, 0.35)	−1.69 (−3.98, 0.61)	−1.29 (−2.92, 0.34)	0				
I-131+PTU	−3.09 (−5.96, −0.22)	−2.29 (−5.64, 1.05)	−2.28 (−5.77, 1.22)	−1.88 (−4.88, 1.12)	−0.59 (−4.00, 2.82)	0			
PTU+PDN	−4.19 (−8.70, 0.32)	−3.39 (−5.55, −1.24)	−3.38 (−5.77, −0.99)	−2.98 (−4.74, −1.23)	−1.69 (−3.95, 0.57)	−1.10 (−4.57, 2.37)	0		
MMI	−3.81 (−7.75, 0.14)	−1.98 (−3.51, −0.45)	−3.67 (−5.38, −1.96)	−3.28 (−3.84, −2.71)	−1.23 (−5.19, 2.73)	1.87 (1.27, 2.47)	−1.39 (−4.44, 1.66)	0	
PTU	−6.36 (−10.59, −2.13)	−5.56 (−7.05, −4.06)	−5.54 (−7.36, −3.73)	−5.15 (−5.97, −4.32)	−3.86 (−5.50, −2.21)	−3.27 (−6.37, −0.16)	−2.17 (−3.72, −0.61)	4.99 (0.39, 9.60)	0

#### FT4

##### Evidence network diagram.

FT4 was reported in 145 RCTS, involving nine therapeutic measures. The network relationship was centered on MMI, and the size of the dots represented the sample size of the interventions, indicating that MMI had the largest number of literatures and the largest sample size. There is a closed loop, so the inconsistency test is carried out, *P* > 0.05, the data basically meet the consistency test. See Fig. 3C for details.

##### SUCRA probability ranking plot.

On FT4, the SUCRA probability sequence diagram is I-131+PTU > I-131+LC > I-131 > MMI+PDN > MMI+PHT > I-131+MMI > PTU+PDN > MMI > PTU, indicating that I-131+PTU is the best way to reduce FT4. See Fig. 4C.

##### League table.

A network meta-analysis was performed on the included studies, and a total of 18 pairings were generated. The results showed that the differences were statistically significant (*p* < 0.05) as follows: I-131+PTU was superior to I-131, MMI+PDN, MMI+PHT, I-131+MMI, PTU+PDN; I-131+LC is superior to MMI+PHT, PTU+PDN, PTU; I-131 is superior to PTU+PDN, MMI, PTU; MMI+PDN is better than MMI, PTU; MMI+PHT is better than MMI, PTU; I-131+MMI is better than MMI, PTU; PTU+PDN is better than MMI, PTU, see Table 3.

**Table 3:** League table of FT3.

Intervention measure	I-131+PTU	I-131+LC	I-131	MMI+PDN	MMI+PHT	I-131+MMI	PTU+PDN	MMI	PTU
I-131+PTU	0								
I-131+LC	−9.91 (−20.29, 0.48)	0							
I-131	−11.64 (−21.05, -2.23)	−1.73 (−6.11, 2.64)	0						
MMI+PDN	−12.68 (−24.57, −0.78)	−2.77 (−10.12, 4.58)	−1.04 (−8.31, 6.24)	0					
MMI+PHT	−14.23 (−24.20, −4.27)	−4.33 (−7.76, −0.89)	−2.59 (−5.86, 0.67)	−1.56 (−8.31, 5.19)	0				
I-131+MMI	−14.70 (−25.31, −4.09)	−4.79 (−9.80, 0.21)	−3.06 (−7.95, 1.83)	−2.03 (−9.69, 5.64)	−0.47 (−4.53, 3.60)	0			
PTU+PDN	−16.76 (−27.35, −6.18)	−6.86 (−11.81, −1.91)	−5.12 (−9.96, −0.29)	−4.09 (−11.72, 3.54)	−2.53 (−6.53, 1.47)	−2.06 (−7.47, 3.35)	0		
MMI	3.66 (2.34, 4.98)	−6.00 (−12.63, 0.62)	−7.04 (−10.04, −4.04)	−18.68 (−28.56, −8.80)	−4.45 (−5.73, −3.17)	18.24 (6.29, 30.20)	−3.98 (−7.84, −0.12)		
PTU	−22.34 (−32.31, −12.37)	−12.43 (−15.88, −8.98)	−10.70 (−13.98, −7.42)	−9.66 (−16.42, −2.91)	−8.11 (−9.94, −6.27)	−7.64 (−11.72, −3.56)	−5.58 (−9.13, −2.02)	5.71 (−4.22, 15.64)	0

#### TSH

##### Evidence network diagram.

TSH was reported in 124 RCTS, involving 9 therapeutic measures. The network relationship was centered on MMI, and the dot size represented the sample size of the intervention, indicating that MMI had the largest number of literatures and the largest sample size. There is a closed loop, so the inconsistency test is carried out, *P* > 0.05, the data basically meet the consistency test. See Fig. 3D for details.

##### SUCRA probability ranking plot.

On TSH, the SUCRA probability sequence diagram is I-131+PTU > I-131 > MMI+PDN > I-131+LC > MMI+PHT > MMI > PTU+PDN > I-131+MMI > PTU, indicating that I-131+PTU increases TSH optimally, as shown in Fig. 4D.

##### League table.

A network meta-analysis was performed on the included studies, and a total of 18 pairwise comparisons were generated. The results showed that the differences were statistically significant (*p* < 0.05) as follows: I-131+PTU was superior to MMI+PHT, I-131+MMI,PTU +PDN,PTU; I-131 is better than MMI+PHT, MMI, PTU+PDN, I-131+MMI, PTU; I-131+LC is superior to PTU; I-131 is superior to PTU+PDN,MMI,PTU; MMI+PDN is better than PTU; MMI+PHT is better than PTU,PTU; MMI is better than PTU. See Table 4.

**Table 4:** League table of TSH.

Interventiuon measure	I-131+PTU	I-131	MMI+PDN	MMI+I-131	MMI+PHT	MMI	PTU+PDN	I-131+MMI	PTU
I-131+PTU	0								
I-131	1.02 (−0.91, 2.95)	0							
MMI+PDN	1.71 (−0.76, 4.19)	0.69 (−0.86, 2.24)	0						
MMI+I-131	2.00 (−0.18, 4.18)	0.98 (−0.03, 1.99)	0.29 (−1.25, 1.82)	0					
MMI+PHT	2.34 (0.25, 4.42)	1.32 (0.53, 2.11)	0.63 (−0.77, 2.02)	0.34 (−0.41, 1.09)	0				
MMI	−2.42 (−4.92, 0.08)	1.39 (0.66, 2.13)	−0.22 (−1.11, 0.68)	−0.10 (−0.89, 0.69)	0.07 (−0.21, 0.36)	0			
PTU+PDN	2.51 (0.30, 4.72)	1.49 (0.42, 2.57)	0.80 (−0.78, 2.38)	0.51 (−0.54, 1.56)	0.17 (−0.67, 1.01)	−0.70 (−2.07, 0.67)	0		
I-131+MMI	2.63 (0.38, 4.88)	1.61 (0.46, 2.76)	0.92 (−0.72, 2.55)	0.63 (−0.50, 1.76)	0.29 (−0.65, 1.23)	−2.41 (−4.48, −0.35)	0.12 (−1.07, 1.31)	0	
PTU	3.12 (1.04, 5.21)	2.11 (1.32, 2.89)	1.41 (0.01, 2.81)	1.13 (0.37, 1.88)	0.79 (0.38, 1.20)	0.71 (0.42, 1.00)	0.61 (−0.12, 1.35)	0.50 (−0.44, 1.43)	0

#### Adverse reactions

##### Evidence network diagram.

Adverse reactions were reported in 89 RCTS, involving nine therapeutic measures. The network relationship was centered on MMI, and the size of the dots represented the sample size of the interventions, indicating that MMI had the largest number of literatures and the largest sample size. There is a closed loop, so the inconsistency test is carried out, *P* > 0.05, the data basically meet the consistency test. See Fig. 3E for details.

##### SUCRA probability ranking plot.

When adverse reactions occur, the SUCRA probability ranking diagram is I-131+PTU > I-131 > I-131+MMI > MMI+PDN > MMI+PHT > MMI > PTU+PDN > I-131+LC > PTU, indicating that I-131+PTU is the best way to reduce the occurrence of adverse reactions. See Fig. 4E.

##### League table.

A network meta-analysis was performed on the included studies, and a total of 18 pair-to-pair comparisons were generated. The results showed that the differences were statistically significant (*p* < 0.05) as follows: I-131+PTU was superior to I-131+LC,PTU,MMI,PTU +PDN,PTU; I-131 is superior to MMI,PTU+PDN; I-131+MMI is superior to MMI,PTU+PDN,PTU; MMI+PHT is better than MMI,PTU; MMI+PDN is better than MMI,PTU; MMI+PHT is better than MMI,PTU; MMI is better than PTU+PDN. See Table 5.

**Table 5:** League table of adverse reactions.

Intervention measure	I-131+PTU	I-131	I-131+MMI	MMI+PDN	MMI+PHT	MMI	PTU+PDN	I-131+LC	PTU
I-131+PTU	0								
I-131	−0.56 (−2.15, 1.03)	0							
I-131+MMI	−0.68 (−2.60, 1.23)	−0.12 (−1.19, 0.95)	0						
MMI+PDN	−0.98 (−2.92, 0.97)	−0.42 (−1.54, 0.70)	−0.29 (−1.37, 0.79)	0					
MMI+PHT	−1.14 (−2.95, 0.66)	−0.58 (−1.43, 0.26)	−0.46 (−1.25, 0.33)	−0.17 (−1.02, 0.69)	0				
MMI	2.20 (0.07, 4.33)	−1.24 (−2.03, −0.46)	−1.80 (−3.58, −0.03)	−1.12 (−1.85, −0.39)	−0.66 (−0.97, −0.35)	0			
PTU+PDN	−2.08 (−4.12, −0.03)	−1.52 (−2.81, −0.23)	−1.39 (−2.65, −0.14)	−1.10 (−2.39, 0.20)	−0.93 (−2.00, 0.14)	0.83 (0.03, 1.62)	0		
I-131+LC	−2.60 (−5.12, −0.09)	−2.05 (−4.00, −0.09)	−1.92 (−3.85, 0.01)	−1.63 (−3.58, 0.33)	−1.46 (−3.27, 0.35)	−0.27 (−1.29, 0.75)	−0.53 (−2.58, 1.53)	0	
PTU	−2.72 (−4.52, −0.91)	−2.16 (−3.00, −1.31)	−2.03 (−2.83, −1.24)	−1.74 (−2.59, −0.89)	−1.57 (−2.01, −1.13)	−0.91 (−1.22, −0.60)	−0.64 (−1.61, 0.33)	−0.11 (−1.92, 1.70)	0

#### Result summary

Based on the network meta-analysis, the combined treatment showed better efficacy than monotherapy. Specifically, the highest total effective rates were observed with I-131+LC, MMI+PHT, and I-131. The most significant reductions in FT3 were achieved with MMI+PDN, I-131+LC, and I-131. The best effects in reducing FT4 were seen with I-131+PTU, I-131+LC, and I-131. The most effective in increasing TSH were I-131+PTU, I-131, and MMI+PDN. The fewest adverse reactions were reported with I-131+PTU, I-131, and I-131+MMI.

### Consistency test

In this NMA, under the consistency model, the PSRF results of all network meta-analyses ranged from 1.00 to 1.05, indicating good convergence.

### GRADE quality evaluation

Evaluation the evidence quality of the outcome indicators using GRADE pro GDT. All the included studies were RCT, so the highest level was preset, and then the downgrade was evaluated according to the degradation factors of the five GRADE specifications. The results are shown as described in Table 6. Each outcome measure was evaluated for GRADE quality of evidence, effective rate, FT3, FT4, TSH, and adverse effects were intermediate quality evidence.

**Table 6:** GRADE quality evaluation.

Outcome	Bias risk	Inconsistency	Indirectness	Inaccuracy	Publication bias	GRADE
Effective rate	Reduce 1 level	Not downgraded (Not Serious)	Not downgraded (Not Serious)	Not downgraded (Not Serious)	Not downgraded (Not Serious)	⊕⊕⊕∘/medium
FT3	Reduce 1 level	Not downgraded (Not Serious)	Not downgraded (Not Serious)	Not downgraded (Not Serious)	Not downgraded (Not Serious)	⊕⊕⊕∘/medium
FT4	Reduce 1 level	Not downgraded (Not Serious)	Not downgraded (Not Serious)	Not downgraded (Not Serious)	Not downgraded (Not Serious)	⊕⊕⊕∘/medium
TSH	Reduce 1 level	Not downgraded (Not Serious)	Not downgraded (Not Serious)	Not downgraded (Not Serious)	Not downgraded (Not Serious)	⊕⊕⊕∘/medium
Adeverse reacion	Reduce 1 level	Not downgraded (Not Serious)	Not downgraded (Not Serious)	Not downgraded (Not Serious)	Not downgraded (Not Serious)	⊕⊕⊕∘/medium

**Notes.**

⊕⊕∘∘Indicates low quality; ⊕⊕⊕∘Mean a medium quality.

## Discussion

### Principal findings of this research

Internationally, the diagnosis of hyperthyroidism mainly relies on laboratory tests and imaging examinations. Laboratory tests usually include the measurement of indicators such as thyroid-stimulating hormone (TSH), free triiodothyronine (FT3), and free thyroxine (FT4). The typical laboratory findings in patients with hyperthyroidism are decreased TSH levels, along with increased FT3 and FT4 levels. In terms of imaging examinations, thyroid ultrasound can be used to determine the location, size, number, and imaging characteristics of thyroid nodules. Color Doppler ultrasound can assess the condition of thyroid blood vessels, which is helpful for distinguishing different types of hyperthyroidism. Thyroid scintigraphy (*e.g.*, I-131 or 99mTcO4- thyroid scintigraphy) can identify different types of hyperthyroidism and provide strategic guidance for I-131 therapy.

The mechanism of hyperthyroidism is complex, and it is currently believed that hyperthyroidism, particularly that caused by Graves’ disease, is associated with autoantibodies that bind to and activate TSHR (Lanzolla, Marinò & Menconi, 2024). Although Graves’ hyperthyroidism is relatively common, there is no standardized treatment protocol available. The established treatment options are antithyroid drugs, I-131, and surgery (Namwongprom, Dejkhamron & Unachak, 2021). In this study, we analyzed the efficacy and safety of oral medications for hyperthyroidism, conducting a detailed analysis of clinical effectiveness rates, FT3, FT4, TSH, and adverse events across multiple drugs for the first time. A total of 3,369 articles were screened, with 151 ultimately included. The risk of bias assessment indicated that the methodological quality of the included studies was moderate, primarily due to the lack of detailed reporting on methods for allocation concealment and blinding.

This network meta-analysis found that compared to monotherapy with a single oral drug, combined treatment with I-131 significantly improved the clinical efficacy in patients with hyperthyroidism. Therefore, the use of I-131 combination therapy for hyperthyroidism is a good choice in clinical practice.

### The main components of drugs and possible mechanisms

In summary, the efficacy and serum index detection mainly focus on I-131, LC, and PTU. In Singer et al. (1995) clearly stipulated that I-131 should be the first-choice treatment for hyperthyroidism. Currently, I-131 is widely used in the United States and Europe. I-131 is an artificial radioactive nuclide with a half-life of 8.3 days. However, I-131 is also a highly toxic nuclide, with the thyroid being its main target organ. Approximately 30% of iodine can be captured by the thyroid, while 70% of iodine is excreted through the kidneys. The β-rays emitted by I-131 can produce ionizing radiation biological effects, which destroy the thyroid follicular epithelium and exert the clinical therapeutic effect (Falk, 1997). Methimazole inhibits the production of new thyroid hormones in the thyroid gland (Li et al., 2018). It works by inhibiting thyroid peroxidase, which typically converts iodide into iodine molecules and incorporates iodine molecules into the amino acid tyrosine. As a result, DIT (diiodotyrosine) and MIT (monoiodotyrosine) are not produced, which are the main components for the synthesis of thyroxine (T4) and triiodothyronine (T3). In the periphery, it acts by inhibiting the conversion of T4 to T3, affecting both the existing thyroid hormones stored in the thyroid and those circulating in the blood. However, it has a short half-life, poor absorption rate, and remains in the body for a long time, which leads to a higher incidence of adverse reactions (Abdi, Amouzegar & Azizi, 2019). Lithium carbonate is rapidly and completely absorbed orally, with peak blood concentrations occurring 2 to 4 h after administration. Lithium ions are initially distributed in the extracellular fluid and then gradually accumulate within cells. They do not bind to plasma proteins, and the half-life (T1/2) is approximately 18 to 36 h. Although lithium is absorbed quickly, it takes some time to cross the blood–brain barrier and enter brain tissue and nerve cells. The use of lithium ions as an adjuvant in the treatment of hyperthyroidism was reported as early as 1976. Kumar et al. (2019) believed that low-dose lithium carbonate could increase the retention of radioactive iodine in the thyroid, thereby enhancing therapeutic efficacy. Therefore, lithium carbonate is often used in combination with I-131 for the treatment of hyperthyroidism. Studies have shown that lithium doses can increase the uptake of I-131 in the thyroid without interfering with the controlled circulating levels of thyroid hormones (Turner, Brownlie & Rogers, 1976).

Thus, despite the challenges in selecting the optimal treatment plan for hyperthyroidism through network meta-analysis, the highest-ranked interventions reveal that I-131 and I-131 combination therapies still have significant advantages in terms of efficacy rates, reducing adverse reactions, and improving related indicators. These interventions can serve as references for clinical medication, but choices should still be made based on the patient’s physical condition and the severity of the disease.

### Possible mechanisms of side effects of drug

In summary, regarding the adverse reaction indicators, the following drugs are better at reducing adverse reactions, with a discussion on I-131, PTU, and MMI. The energy released by I-131 within thyroid tissue can destroy thyroid follicular epithelial cells, reducing the synthesis and secretion of thyroid hormones. If the degree of thyroid cell damage is too severe after treatment and the thyroid tissue cannot compensatorily maintain normal thyroid function, this can lead to hypothyroidism, radiation thyroiditis, and damage to the reproductive and hematopoietic systems. Some patients may experience these effects shortly after treatment, while others may develop them months or even years later (Brownlie, Turner & Ovenden, 1979). When using I-131 for treatment, doctors will comprehensively assess the patient’s condition and physical status, weigh the pros and cons of the treatment, and take corresponding measures to prevent and manage potential side effects. Methimazole (MMI) may suppress bone marrow hematopoietic function, leading to leukopenia, and in severe cases, agranulocytosis. This occurs because the drug and its metabolites may act as haptens, binding with proteins in the body to form antigens that trigger an immune response (Abraham & Acharya, 2010). This response generates antibodies against hematopoietic cells, affecting the production, maturation, and release of granulocytes in the bone marrow, thereby reducing the number of white blood cells in the peripheral blood. Additionally, methimazole may interfere with the normal function of hematopoietic stem cells, affecting cell proliferation and differentiation, leading to other hematological abnormalities such as thrombocytopenia. MMI inhibits the activity of thyroid peroxidase, reducing the synthesis of thyroid hormones. If the dosage is too high or the duration of use is too long, it may excessively inhibit the synthesis of thyroid hormones, leading to hypothyroidism. At this point, with reduced thyroid hormone levels and weakened negative feedback regulation, the pituitary gland increases the secretion of TSH, which can cause compensatory thyroid hyperplasia. PTU and its metabolites may act as haptens, binding with proteins in the body to form antigens that stimulate the immune system to produce antibodies. These antibodies can bind to hematopoietic stem cells or mature granulocytes in the bone marrow, leading to reduced granulocyte production and increased destruction, thereby causing leukopenia and, in severe cases, agranulocytosis (National Library of Medicine, 2020). This immune-mediated damage can interfere with normal hematopoietic function in the bone marrow, reducing the number of white blood cells in the peripheral blood and weakening the body’s ability to fight infections. During the metabolism of PTU in the liver, some hepatotoxic metabolites are produced. These metabolites can directly bind to biomacromolecules within hepatocytes (such as proteins and nucleic acids), disrupting the normal structure and function of liver cells and leading to hepatocyte degeneration and necrosis. By inhibiting the activity of thyroid peroxidase, PTU reduces the synthesis of thyroid hormones. If the dosage is too high or the duration of use is too long, it can excessively inhibit the synthesis of thyroid hormones, leading to hypothyroidism. A study (Yoshihara et al., 2019) comparing the effectiveness of MMI and PTU in a multicenter cohort found no significant differences in mortality or adverse events between patients treated with PTU or MMI.

### Outlook on the prospects of biological agents

The focus will be on novel therapies such as biological agents, and their value as future research directions for hyperthyroidism will be briefly elaborated from aspects including mechanism of action, research progress, and future potential, with emphasis on their innovativeness and application prospects.

### Biological agents targeting TRAb

Targeting TRAb, the core pathogenic factor of Graves’ disease, monoclonal antibodies (*e.g.*, rituximab, teplizumab) exert their effects by eliminating B cells that produce TRAb or blocking the binding of TRAb to thyroid-stimulating hormone receptors (Marinò et al., 2014). Currently, Phase II clinical trials have shown that these antibodies can reduce TRAb levels and alleviate hyperthyroidism symptoms. In the future, it is necessary to explore and optimize administration regimens and investigate long-term efficacy.

### Novel immunomodulators

Small-molecule inhibitors (*e.g.*, JAK inhibitors) targeting Th1/Th2 immune imbalance, and biological agents that regulate the function of Treg cells are expected to control the progression of Graves’ disease from the immune source. Relevant basic research has confirmed their immunomodulatory potential, and accelerated translation to clinical practice is required (Lee et al., 2023).

### Novel therapies targeting thyroid nodules

For toxic multinodular goiter and autonomous hyperfunctional thyroid adenomas, novel ultrasound-guided ablation techniques combined with targeted drugs (*e.g.*, small-molecule inhibitors that inhibit nodule proliferation) may replace traditional surgery or radioactive iodine therapy. Currently, early clinical studies have shown high safety, and further verification of long-term efficacy is needed.

These novel therapies provide new ideas for the precise treatment of hyperthyroidism. In the future, it is necessary to clarify their effectiveness and safety through multi-center clinical trials, and optimize treatment regimens in combination with the concept of personalized medicine.

The geographical difference in hyperthyroidism treatment, namely “I-131 as the mainstay in North America and ATD as the core in Europe and Asia”, is essentially the result of the combined effects of medical systems, patient needs, and clinical concepts. Based on data from international multicenter studies conducted between 2013 and 2023, the historical background of the high application rate of I-131 in North America is closely related to medical cost control and patient compliance requirements: the single-treatment nature of I-131 can reduce the consumption of medical resources associated with long-term follow-up and multiple medication administrations, while also meeting the demand of some patients for a “simplified treatment process”. However, this trend has undergone a significant shift over the past 20 years. A cohort study on GD treatment in North America, published in the Journal of Clinical Endocrinology & Metabolism in 2021, showed that the application rate of I-131 decreased from 65% in 2000 to 42% in 2020, with a corresponding increase in the application rate of ATD. The core reason lies in the deepened clinical understanding of the long-term management costs of lifelong hypothyroidism caused by I-131 and its impact on patients’ quality of life. Lifelong hypothyroidism requires continuous levothyroxine replacement therapy, which instead increases long-term medical burdens and patients’ dependence on medication. This has led North American clinicians to prefer reversible treatment options such as ATD.

The selection of ATD as the first-line treatment in Europe and Asia reflects the accurate alignment with the characteristics of patient populations and treatment goals. Both the Pacini et al. (2022) and China Association of Integrative Medicine, China Association of Chinese Medicine, Chinese Medical Association (2023) emphasize that the proportion of adolescent GD patients is higher in Europe and Asia. Such patients have a stronger demand to “avoid lifelong medication”. Through the three-phase treatment of “control—tapering—maintenance”, ATD is expected to achieve immune remission, which is in line with the clinical pursuit of “functional cure”.

Notably, this review overly focuses on the efficacy review of traditional treatment regimens and fails to incorporate breakthrough studies on the autoimmune mechanism of GD in the past 5–10 years, which constitutes a core limitation (Lee et al., 2023). As pointed out, “mechanistic therapies such as immunosuppressants and monoclonal antibodies represent the future direction”. Monoclonal antibodies targeting the TRAb pathway (*e.g.*, rituximab) and new immunomodulators have shown potential in targeted regulation of autoimmune disorders in early clinical trials. The omission of this field in this review makes it unable to reflect the forefront of clinical research and fails to provide a reference for innovative treatment options in clinical practice (Braverman & Pearce, 2025). Future studies need to address this gap by systematically integrating evidence-based data on mechanistic therapies to provide more comprehensive guidance for clinical decision-making (Azizi et al., 2024).

### Limitation

This study is Network Meta-Analysis (NMA) based on frequentist methodology, which has certain guiding significance for evaluating the effectiveness and safety of different drug treatments for hyperthyroidism. In order to accurately assess their efficacy and safety and promote their application in actual clinical practice, it is necessary to further improve the methodological quality of randomized controlled trials. Therefore, more rigorously designed randomized controlled trials are needed in the future to further explore the effectiveness and safety of oral medications for hyperthyroidism. In addition to clinical efficacy, the safety of oral medications is also particularly important. Thus, further experimental and clinical evidence is required to verify the safety of these medications. A research system, as well as post-marketing safety monitoring and reassessment, should be established. We will emphasize that due to the diversity of patient characteristics, these findings may not be applicable to all patients. Therefore, in clinical practice, the selection of appropriate treatment regimens should be based on the specific circumstances of individual patients. For future research, in order to further enhance the generality and applicability of studies, we recommend that future research designs and implementations should fully consider the differences in patient characteristics, and adopt methods such as stricter inclusion criteria and stratified analysis to reduce the impact of bias and confounding factors.

## Conclusions

In summary, the current evidence suggests that combination therapy for hyperthyroidism is often more effective than therapy alone. Among them, the effective rate of I-131+LC is better than other treatment measures, I-131+PTU is better than other treatment measures in reducing FT3, increasing TSH and reducing adverse reactions. Due to the limitations in the number and quality of the included studies, the above conclusions await further verification from more large-sample, high-quality, and multicenter studies. This article aims to provide a new way of thinking for clinical treatment of hyperthyroidism.

## Supplemental Information

10.7717/peerj.20403/supp-1 Supplemental Information 1 PRISMA checklist

10.7717/peerj.20403/supp-2 Supplemental Information 2 Pubmed Search strategy

10.7717/peerj.20403/supp-3 Supplemental Information 3 The basic information of the included literature

## Abbreviation

I-131: Iodine-131
MMI: Methimazole
PDN: Prednisone
PTU: Propylthiourailc
LC: Lithium carbonate
PHT: Propranolol
T3: Triiodothyronine
T4: Thyroid hormone
TgAb: Thyroglobulin antibodies
TSH: Thyroid stimulating hormone
FT3: Free triiodothyronine
FT4: Free thyroxine
TSHR: Thyroid-stimulating hormone receptor
TPOAb: Thyroid peroxidase antibody
TgAb: Thyroglobulin antibody
ATDs: Antithyroid drugs

## Additional Information and Declarations

### Data Availability

The following information was supplied regarding data availability: The raw data are available in the Supplemental File.
