# Supplementary material for: Comparing efficacy and safety of oral drugs in treatment of hyperthyroidism: a systematic review and network meta-analysis
Source: PeerJ. 2026 Jan 28;14:e20403. doi: 10.7717/peerj.20403 (PMC12860280; doi:10.7717/peerj.20403)
Supplement: Supplemental Information 2 [file peerj-14-20403-s002.docx]

Pubmed Search Strategy

| Order | Strategy |
| --- | --- |
| #1 | Search: "Hyperthyroidism"[Mesh] |
| #2 | Search:(((Hyperthyroid[Title/Abstract]) OR (Hyperthyroids[Title/Abstract])) OR (Primary Hyperthyroidism[Title/Abstract])) OR (Hyperthyroidism, Primary[Title/Abstract]) |
| #3 | Search:#1 OR #2 |
| #4 | Search:"Propylthiouracil"[Mesh] |
| #5 | Search:(6-Propyl-2-Thiouracil[Title/Abstract]) OR (6 Propyl 2 Thiouracil[Title/Abstract]) |
| #6 | Search:"Chlorotoxin" [Supplementary Concept] |
| #7 | Search:(TM601 peptide[Title/Abstract]) OR (TM 601[Title/Abstract])) OR (TM-601[Title/Abstract])) OR (131I-TM-601[Title/Abstract])) OR (chlorotoxin I-131[Title/Abstract]) |
| #8 | Search: "Methimazole"[Mesh] |
| #9 | Search: (Methimazole[Title/Abstract]) OR (Methymazol[Title/Abstract])) OR (1-Methyl-2-mercaptoimidazole[Title/Abstract])) OR (1 Methyl 2 mercaptoimidazole[Title/Abstract])) OR (Merkazolil[Title/Abstract])) OR (Methylmercaptoimidazole[Title/Abstract])) OR (Thiamazole[Title/Abstract])) OR (Thimazol[Title/Abstract])) OR (Mercasolyl[Title/Abstract])) OR (Tiamazol[Title/Abstract])) OR (Mercazolyl[Title/Abstract])) OR (Tapazole[Title/Abstract])) OR (Metizol[Title/Abstract])) OR (Mercazol[Title/Abstract])) OR (Mercazole[Title/Abstract])) OR (Favistan[Title/Abstract])) OR (Favistan[Title/Abstract])) OR (Methizol[Title/Abstract])) OR (Strumazol[Title/Abstract])) OR (Thiamazol Henning[Title/Abstract])) OR (Henning, Thiamazol[Title/Abstract])) OR (Thiamazol Hexal[Title/Abstract])) OR (Hexal, Thiamazol[Title/Abstract])) OR (Thyrozol[Title/Abstract]) |
| #10 | Search:"Propranolol"[Mesh] |
| #11 | Search:(((((((((((((Propanolol[Title/Abstract]) OR (AY-20694[Title/Abstract])) OR (AY 20694[Title/Abstract])) OR (Propranolol Hydrochloride[Title/Abstract])) OR (Hydrochloride, Propranolol[Title/Abstract])) OR (Inderal[Title/Abstract])) OR (Avlocardyl[Title/Abstract])) OR (Obsidan[Title/Abstract])) OR (Obzidan[Title/Abstract])) OR (Dociton[Title/Abstract])) OR (Betadren[Title/Abstract])) OR (Dexpropranolol[Title/Abstract])) OR (Anaprilin[Title/Abstract])) OR (Anapriline[Title/Abstract]) |
| #12 | Search:"Lithium Carbonate"[Mesh] |
| #13 | Search:((((((((((((((Carbonate, Lithium[Title/Abstract]) OR (Dilithium Carbonate[Title/Abstract])) OR (Carbonate, Dilithium[Title/Abstract])) OR (Priadel[Title/Abstract])) OR (Lithonate[Title/Abstract])) OR (CP-15,467-61[Title/Abstract])) OR (CP 15,467 61[Title/Abstract])) OR (CP15,46761[Title/Abstract])) OR (Lithotabs[Title/Abstract])) OR (Lithobid[Title/Abstract])) OR (Lithane[Title/Abstract])) OR (Eskalith[Title/Abstract])) OR (Micalith[Title/Abstract])) OR (Lithium Bicarbonate[Title/Abstract])) OR (Bicarbonate, Lithium[Title/Abstract]) |
| #14 | Search:"Prednisone"[Mesh] |
| #15 | Search:(Dehydrocortisone[Title/Abstract]) OR (delta-Cortisone[Title/Abstract])) OR (Cortancyl[Title/Abstract])) OR (Panafcort[Title/Abstract])) OR (Deltasone[Title/Abstract])) OR (Encortone[Title/Abstract])) OR (Encorton[Title/Abstract])) OR (Liquid Pred[Title/Abstract])) OR (Meticorten[Title/Abstract])) OR (Pronisone[Title/Abstract])) OR (Ultracorten[Title/Abstract])) OR (Apo-Prednisone[Title/Abstract])) OR (Cortan[Title/Abstract])) OR (Decortin[Title/Abstract])) OR (Dacortin[Title/Abstract])) OR (Decortisyl[Title/Abstract])) OR (Orasone[Title/Abstract])) OR (Panasol[Title/Abstract])) OR (Predni Tablinen[Title/Abstract])) OR (Prednidib[Title/Abstract])) OR (Prednison Acsis[Title/Abstract])) OR (Acsis, Prednison[Title/Abstract])) OR (Prednison Galen[Title/Abstract])) OR (Prednison Hexal[Title/Abstract])) OR (Rectodelt[Title/Abstract])) OR (Sone[Title/Abstract])) OR (Sterapred[Title/Abstract])) OR (Winpred[Title/Abstract]) |
| #16 | Search:#4 OR #5 OR #6 OR #7 OR #8 OR #9 OR #10 OR #11 OR #12 OR #13 OR #14 OR #15 |
| #17 | Search:((Randomized controlled trial[Title/Abstract]) OR (Randomized[Title/Abstract])) OR (Placebo[Title/Abstract]) |
| #18 | Search:#3 AND #16 AND #17 |
